# Teaching computational systems biology with an eye on quantitative systems pharmacology at the undergraduate level: Why do it, who would take it, and what should we teach?

**DOI:** 10.3389/fsysb.2022.1044281

**Published:** 2022-11-25

**Authors:** Ioannis P. Androulakis

**Affiliations:** 1 Biomedical Engineering Department, New Brunswick, NJ, United States; 2 Chemical and Biochemical Engineering Department, Rutgers University, New Brunswick, NJ, United States

**Keywords:** computational systems biology, computational systems pharmacology, engineering, biomedical, undergraduate

## Abstract

Computational systems biology (CSB) is a field that emerged primarily as the product of research activities. As such, it grew in several directions in a distributed and uncoordinated manner making the area appealing and fascinating. The idea of not having to follow a specific path but instead creating one fueled innovation. As the field matured, several interdisciplinary graduate programs emerged attempting to educate future generations of computational systems biologists. These educational initiatives coordinated the dissemination of information across student populations that had already decided to specialize in this field. However, we are now entering an era where CSB, having established itself as a valuable research discipline, is attempting the next major step: Entering undergraduate curricula. As interesting as this endeavor may sound, it has several difficulties, mainly because the field is not uniformly defined. In this manuscript, we argue that this diversity is a significant advantage and that several incarnations of an undergraduate-level CSB biology course could, and should, be developed tailored to programmatic needs. In this manuscript, we share our experiences creating a course as part of a Biomedical Engineering program.

## Introduction

We wish to begin this discussion by stating that this work is neither a review of systems biology courses nor a generic template of what a “system biology course” should be. Instead, in this manuscript, we wish to share one researcher/instructor’s motivation and struggles when “developing” a computational systems biology (CSB) course and why and how this researcher/instructor envisioned and implemented the development of such a course.

First, when developing any course, it is essential to ask a critical question: What is the topic covered by the course? The curriculum of any academic department, and at this point, we wish to emphasize that we are focusing on undergraduate education, is developed around critical competencies the graduates of the program need to master.

These key competencies, in the parlance of a curriculum, are developed through the teaching of “core courses” that graduates of a specific major are expected to master to a reasonable level (in addition to general education and discipline-specific elective courses). These courses come together, almost in the form of a 3-days hierarchical puzzle, with one layer as the foundation of the next. At the same time, each layer is also composed of interlocking elements (courses). So, taking my home department (Biomedical Engineering, BME) as an example, in their freshman year, students take mostly math, physics, chemistry, and biology courses, often in fall/spring sequences, thus forming a foundation for the sophomore year where students are introduced to biomedical engineering principles and human physiology using 1st-year courses as the foundation and building bridges between BME and physiology/biology. This leads to the 3rd year with a focus on BME thermodynamics, kinetics, transport, devices, biomaterials, and numerical analysis, all of which depend on earlier courses and feeding off each other. Eventually, students in their 4th year focus on senior design and BME electives, which draw from the foundations of earlier years.

In developing a curriculum, and by extension, courses, the material taught has to be correctly placed in a context that is defined based on the concept of subject matter. Thus, the subject matter becomes central in developing a course and needs to meet two broad criteria: First, the scope of the subject matter needs to be clearly articulated; and second, different subject matters need to rationally inter-connect so that, eventually, a discipline can emerge. Let us consider a typical BME course, “biomedical transport phenomena.” Although the way the material is covered can substantially differ from institution to institution (even from instructor to instructor), the course description will inevitably boil down to something like “[the course] introduces and applies the concepts of momentum, mass, and thermal energy transport in the context of problems of interest in biomedical sciences and engineering^1^.” In fact, it is interesting that a very similar description would also apply to my other academic home, Chemical Engineering, with a slight change of the focus of the applications. We should expect descriptions similar to “[transport phenomena] provides a unified treatment of momentum, mass, and energy transport in chemical engineering problems^2^.” We notice that both course descriptions rely on a common foundation while differing in the scope of the applications. Therefore, the fundamentals on which a course, like “transport phenomena,” is based and the essential content to be delivered are universal, well-defined, and generally agreed upon. The delivery or focus may differ; however, there is no ambiguity regarding the aims, scope, and principles. The same holds for, basically, all courses that define the “core” component of any curriculum.

This brings us to computational systems biology. When thinking about developing a CSB course, the first question one asks is, “what is systems biology?”^3^ As one can imagine, this is an important question because its answer will eventually determine the content of the course. Without delving into the history and origins of the field–well above and beyond the scope of the present manuscript–we quickly realize that defining SB, let alone CSB, is no easy task. This is best exemplified by NIH’s attempt to provide a lay person’s definition of the field, which starts as follows: “Ask five different astrophysicists to define a black hole, the saying goes, and you’ll get five different answers. But ask five biomedical researchers to define systems biology, and you’ll get 10 different answers or maybe more. Systems biology is an approach in biomedical research to understanding the larger picture—be it at the level of the organism, tissue, or cell—by putting its pieces together. It is in stark contrast to decades of reductionist biology, which involves taking the pieces apart.^4^” This attempt to define SB immediately raises a critical concern: defining the term brings to mind the infamous quote, “I shall not today attempt further to define the kinds of material I understand to be embraced within that shorthand description, and perhaps I could never succeed in intelligibly doing so. But I know it when I see it, and the motion picture involved in this case is not that” used in 1964 by United States Supreme Court Justice Potter Stewart to describe his threshold test for obscenity in Jacobellis v. Ohio, and second the idea that SB is focused on looking at the big picture by putting “pieces” together.

A definition succinctly combining the fundamental concepts was provided by the Institute of Systems Biology (ISB), a pioneering institution in the field, suggesting that “Systems biology is based on the understanding that the whole is greater than the sum of the parts.^5^” This brings us to Computational Systems Biology, best described as the application of modeling and computational methods (mathematics, physics, engineering, and computing) for placing holistic systems biology data (the parts of the sum) in a context, extracting quantitative information and making quantifiable predictions based on a wealth of information. We, therefore, distinguish between systems biology and computational systems biology^6^ (SB vs. CSB) in that the latter focuses primarily on the computational or quantitative methods for analyzing SB data, as opposed to the experimental methods and techniques that, by taking a holistic view, generate system-wide observations of the biological system.

Holistic computational approaches, also known as systems-based approaches, are not new, especially to the engineering field (Pistikopoulos et al., 2021). However, it must be noticed that systems-based approaches originated and have been primarily developed to address questions in complex engineered systems, CoES. Systems biology, however, emergence as a result of the need to study complex biological systems, CoBS, (Androulakis, 2014; Androulakis, 2015). Among the many differences between CoES and CoBS, two stand out prominently: First, in CoES the parts making the “whole” are well known and have been defined and designed by the user; second, how the elements interact with each are known, since they have been engineered. Although emergence can be a property of engineered systems (Soria Zurita and Tumer, 2017) it can be analyzed given that the degrees of freedom and the rules are better understood.

On the other hand, in CoBS neither the parts not the way the elements interact are well understood. Emergence in CoBS, which shapes their behavior, is yet to be characterized (Hao et al., 2021). This lack of foundational approaches makes analyzing the “whole” that SB promises challenging.

Which brings us back to our original question: how does one develop a course focusing on the theory, methods, and approaches for studying the “whole” when neither the parts nor the rules that govern the dynamics, and interactions, of the parts are fully understood? CSB emerged due to a lack of first principles understanding of biology, and life, leading to the observation that amassing carefully designed observational data, combined with reverse-engineering approaches, would lead to a reconstruction of the physical/biological/physiological reality. So CSB can be perceived as the compendium of theoretical, mathematical modeling and computational^7^ approaches that will enable us to extract approximations of the fundamental laws to rationally interpret the observations and make well educated predictions. However, because of the enormous complexity of biological systems and the diversity of modalities in which biological and physiological data exist, the types of theory, modeling and computational approaches that can be used form an, almost, uncountable set! Which brings us to heart of the question: how can one design “a” course to capture this enormous diversity?

## 1 Why, who, and what

In designing a course, three questions need to be addressed at the very beginning: Why would the material be of interest to key stakeholders, i.e., students and future employers; who are the students the course will be offered to, i.e., what is their background and broader interests; and finally, what will the students be taught, i.e., which topics of the subject in question will be covered? Regarding CSB, none of these questions are easy to answer. In this direction, it is also essential to make two critical observations: 1) Our emphasis is on computational systems biology (CSB), which implies that the focus is on theoretical, mathematical, and computational methods for analyzing and, especially, modeling biological data; and 2) we aim to address undergraduate students, that is students who have, at best, a solid understanding of their degree-specific fundamentals. The later point is crucial and will be further discussed shortly.

### 1.1 Why teach a course in computational systems biology?

Nobel Laureate David Baltimore 20 years ago stated that because of profound scientific and technological advances, “biology became an information science” (Baltimore, 2001). As we began to probe biological systems at multiple scales of physiological organization, at increased spatial and longitudinal resolution as well as at a population level and started amassing large amounts of diverse information from the cellular to the host and eventually population levels, it became apparent that to upgrade the information content of biological data, mathematical and computer-based methodologies were required (Stephanopoulos, 1999). Therefore, the scientific community has long realized that as the rate of generating biological information increases, the role of CSB becomes critical for translating data and information into to actual knowledge, targets, and actions.

Furthermore, the possibility of incorporating mathematical and computational modeling in business and regulatory decisions is what builds momentum for deploying further systems approaches (Androulakis, 2022). Recently, the FDA made the development of computational modeling a key priority in supporting regulatory decision-making (US FDA, 2011), aiming to facilitate faster, cheaper, and better pathways to market (Drager and Palsson, 2014). FDA’s strategic plan (FDA, 2011) advocated the development of *in silico* computational methods to advance virtual clinical trials connecting individual patient characteristics to outcomes; develop computer models simulating cells and organs to predict safety and efficacy; and integrate modeling with safety data to better predict patient-specific clinical risk-benefit. FDA’s support led to the development of the “Model-informed Drug Development” pilot program^8^ to initiate discussions between regulators and sponsors. A recently concluded scientific exchange and review of clinical drug applications in various therapeutic areas (Bai et al., 2021) revealed a wide range of systems-based approaches for model development, from *de novo* models to adaptation of existing ones; as well as the use of a variety of computational methods, strategies and data modalities by the pharmaceutical industry. Similarly, the European regulatory agencies (European Medicines Agency, EMA) have argued that the value of *in silico* methods in development and evaluation has been convincingly demonstrated and that modeling and simulation has evolved from “being a possible alternative” to a “must for development of new medicines” (Musuamba et al., 2021).

It is, therefore, clear that the need for providing education around CSB exists; it is only expected to grow; and it will serve not only the academic and research communities but also the pharmaceutical industry and the regulatory agencies in making business and approval decisions, with the expectation that demand in this broader domain is only expected to increase. The latter may be the main driver since the broader pharmaceutical sector is the leading employer of college graduates in life sciences, including biomedical (and chemical) engineering.

### 1.2 Who should take a computational systems biology course?

The number of systems biology courses and programs is increasing across the world. A simple search would produce numerous hits. However, one must also observe that most of these programs are either graduate (Ph.D.) or interdisciplinary programs, and often CSB courses are offered as a graduate or high-level undergraduate electives. The implication is that an implicit self-selection process is in place, and students, especially undergraduates, who gravitate towards these courses do so because they have already developed a strong interest and the necessary background. The challenge we faced in developing a CSB course was driven by our desire to introduce a more general population of students to concepts of CSB to expand their arsenal and improve their professional prospects.

However, CSB emerged by the need to bring together diverse disciplines, including mathematics, engineering, and computational sciences, to handle data and address questions arising from life-science fields, such as cell and molecular biology, neuroscience, pharmacology, physiology, and medicine. So, unlike other subjects, which can be more readily associated with a single discipline, in CSB “beauty is in the eyes of the beholder.” SB, and by extension CSB, is the “emergent” result of scientific explorations rather than the attempt to establish a discipline-specific foundation. So, unlike subjects that form the foundation of a “degree” and whose fundamental principles are widely accepted and agreed upon (see the Transport Phenomena example discussed earlier), CSB emerged in an asynchronous and distributed manner while various researchers were attempting to rationalize data and information that was generated in diverse groups during their research activities. As such, CSB is the result of an environment characterized by high entropy levels!

This creates issues because it is not clear how to design a course to match student background on the one hand and the broad spectrum of questions raised under a CSB umbrella on the other.

### 1.3 What to teach in a computational systems biology course

I had my first exposure to CSB about 15 years ago. At the time, my experimental collaborator, an “old fashioned, hardcore, card-carrying” biologist, described to me his first microarray experiments as follows “it felt like I had spent my entire life looking at a tree in my backyard while sitting on my porch and suddenly I climbed the roof of my house and realized I could see the forest.” This is, I believe, a very nice description of SB, especially as we merge SB with pharmacology and physiology into what is now referred to as quantitative systems pharmacology (Vodovotz et al., 2013; Androulakis, 2015; Rao et al., 2016a; Scheff et al., 2016; Putnins et al., 2022). Looking beyond the tree closest to us, not only at all the other trees but also at all other living and nonliving things one can find in a forest, and realizing how they depend on each other, changes our perspective. But once we realized there is a forest, we also realized that there are several ways to look at and explore it.

It is reasonable to assume that the concept of SB may have existed in people’s minds for many years (Mesarovic, 1968); however, it was not until systematic and systemic perturbations became readily available (Ideker et al., 2001) that this realization was appreciated. We then recognized that the biological “forest” could be viewed from different angles, depending on the perturbation, i.e., point of view. This made us move beyond the correlational or simple(r) modeling approaches, which would suffice when the amount of data was easily manageable or the hypothesis well defined, to more advanced methods. However, looking at a problem from different angles, depending on the type of question one asks, results in fundamentally different approaches for analyzing the observations.

Unlike more “traditional” disciplines and subjects, systems biology evolved along with technological advances, enabling the generation of data modalities of increased complexity. Being an evolving endeavor, the more questions were answered, the more questions were generated. One can look at events at the level of the transcriptome, the proteome, the metabolome, or the epigenome to the exposome. One can consider individual signaling cascades or networks within and across cells and organs. We can consider deterministic or stochastic dynamics, discrete or continuous, equation or rule-based, and more recently, machine learning and AI. The data can be numerical, textual, or interpreted. And the list goes on and on (Scheff et al., 2016). The complexity in nicely described in (Klipp et al., 2016), outlining a comprehensive, albeit not exhaustive, list of topics assembled in an attempt to construct an inclusive “Systems biology textbook.”

However, what further complicates “teaching” of systems biology at the undergraduate level is that the methodologies require a solid theoretical, mathematical, and computational foundation, or at least a good understanding of basic concepts and methods. One can readily appreciate the need for some basic statistics and probability; fundamentals of ordinary differential equations, dynamics, and stability; linear algebra; basic concepts of network theory; optimization, which will serve as the foundation for more specific topics from parameter estimation to machine learning; a certain level of familiarity with basic programming concepts along the lines of (Kernighan and Pike, 1999); good understanding with a programming environment/language such as MATLAB, R, Python; basic concepts of numerical analysis, primarily ODE integration; and the list can go on and on. And, of course, we have purposely left out any biological, physiological, and pharmacological background that is needed to at least appreciate the origin, role, and information content of the data that is to be analyzed and modeled!

Of course, it is inconceivable to design a one-semester course that will discuss in depth all the subjects. It would be hard enough to compress each of them into one course, let alone cover them all in one semester or term and do so at the undergraduate level while students develop foundations.

And then comes yet another complication: By definition systems biology is an interdisciplinary topic that we wish to introduce to students of various backgrounds. Therefore, the “one size fits all” approach is bound to fail! Unlike the “transport phenomena” example we discussed earlier, where both chemical and biomedical engineers share a common engineering foundation on which discipline-specific variations can be developed, a common underlying structure does not exist in CSB. Instead, students of different backgrounds (mathematics, computer science, engineering, life science, medicine) have mastered building blocks but do not necessarily have an appreciation for the broader topics or how they come together.

Despite, or maybe inspired by, these difficulties several outstanding attempts have been made to “formalize” CSB by assembling critical information in the form of a standalone textbook to be used either in a classroom or as an independent study guide. There have been many excellent attempts, and we will not provide a comprehensive list here. Instead, a few examples will be used to illustrate some key points^9^. It is interesting to note a sequence authored by a pioneer in the field, Bernhard Palsson. His 2006 book (Palsson, 2006), motivated by his outstanding work on metabolic flux analysis, focused the discussion on the analysis and reconstruction of metabolic networks, primarily emphasizing their steady-state properties. His 2011 book (Palsson, 2011) considers the description of dynamic states; therefore, kinetic aspects of metabolism play a central role. The combination of the two leads to the emergence of network dynamics which is the theme of several equally impressive attempts. The excellent book by Marcus Covert (Covert, 2015) aims to expand on dynamics and presents the material in two sections. The first mainly focuses on methodological discussions of topics such as control theory, Boolean representations, solutions of ODEs, graphical analysis, numerical integration, and stochastic simulation. The first part, therefore, is essentially describing a set of tools. The second section takes a closer look at how these tools enable the analysis of transcriptional regulation, signal transduction, and metabolism modules and introduces the students to mode complex models. The approach of Eberhard Voit, a pioneer in developing mathematical models in biology (Voit, 2018), further expands the modeling tools by presenting a broader introduction to mathematical modeling, including parameter estimation, sensitivity analysis, and population dynamics. Then moves into the modeling of specific sub-systems (from genes to signaling to proteins to metabolism) but then concludes by extending the envelope towards physiological systems and applications of CSB modeling in pharmacology and medicine. Thus, the horizons of the possible applications of CSB are further extended. The final example we will discuss is the book by Alon (2001) which takes a fascinating view of how to approach, and teach, CSB in that it focuses on basic building blocks, i.e., motifs, and shows how these structures (feedforward, feedback, etc.) can lead to the emergence of interesting dynamics (biostability, memory, oscillations, robustness, optimality) and how this modularity guides and underlies function. So, this approach is focused more on design principles.

So, it is fascinating to see how the “blind men and an elephant^10^” parable plays in the field of CSB and how the “holistic” approach interestingly leads to different points of view depending on how one wishes to view the whole. This is not bad; it simply emphasizes the synthetic nature of the field and that in SB we attempt to reconstruct a physical reality based on observations whose nature is complementary instead of fundamental, making the field attractive!

This diversity focuses on the actual protagonist: The course instructor. We believe that instructor preferences could shape the content and focus of a CSB course. This should not be perceived as a drawback or limitation but rather a welcome. The field is rich and should remain diverse, and the practitioners need to bring the required diversity to maintain a robust, holistic approach.

## 2 One approach to teaching computational systems biology with an eye on quantitative systems pharmacology

Quantitative Systems Pharmacology (QSP) is the result of the coming together of four disciplines: 1) Systems biology, 2) systems pharmacology, 3) systems physiology, and 4) data science (Allerheiligen et al., 2011; Azer et al., 2021), all under the umbrella of dynamic systems theory (Chae, 2020). The aim is to develop integrated, multi-scale models predictive of treatment response (Androulakis, 2015; Rao et al., 2016a; Scheff et al., 2016). CSB is one of the closest relatives to QSP (Ghosh et al., 2011) since the latter integrates pharmacology, physiology, and data science using principles, concepts, and computational approaches initially developed under the CSB formalism for describing events at the cellular and molecular levels.

In our attempt to address the needs of a population, primarily undergraduate biomedical engineers, and better prepare them for an evolving workforce; we wished to develop a systematic way to expose students to CSB concepts with “an eye” on QSP. In designing the sequence of themes to be discussed, we wished to remain grounded in reality, realizing that the material and approach were directed toward a student population that had.

- Reasonable familiarity, including elements of linear algebra, calculus, and differential equations, as expected of a biomedical engineering student.
- Good familiarity with very basic biology concepts, as expected of a biomedical engineering major.
- Reasonable familiarity with physiology principles, as expected of a biomedical engineering major.
- Limited, if any, pharmacology background.
- Little, if any, knowledge and/or exposure to computational sciences (especially machine learning).
- Limited understanding of dynamic theory and analysis concepts (such as bifurcation, stability analysis, etc.).
- Little, if any, experience in developing dynamic models beyond what would be discussed in a typical undergraduate-level biomedical engineering course.
- Limited, if any, understanding of optimization, which inevitably will extend to non-linear regression and model building in general.
- Little familiarity with numerical computing beyond topics covered in a typical biomedical engineering numerical modeling course.
- Limited familiarity with computer programming in environments such as Matlab, R, or Python; and last but not least.
- The audience of the course is expected to be students who neither have prior knowledge and experience with the development of mathematical and computational models, let alone CSB, nor have identified this field as one in which they wish to excel in the future.

In other words, we wish to treat this as a typical biomedical engineering course where students develop essential skills and reach a general audience providing them with enough information to spark their interest and establish a foundation they could further build. The course is designed so that it is offered to seniors in Biomedical (or Chemical) Engineering and is a typical 3-credit, semester-long course. This course is currently offered as a stand-alone course providing students with the required foundation to pursue advanced (graduate) coursework.

The above attributes, as earlier discussed, are fundamental because usually, when designing and delivering a CSB course, it is implicitly assumed that students are either experts in some/all quantitative aspects or have a strong interest and/or desire to develop this competency. Here, we wanted to expose students and, if not creating an interest and desire to pursue the field, at least provide enough information so that they could either be engaged in meaningful discussions in future professional interactions or appreciate that CSB could be a likely avenue for addressing questions and problems in the future. It was, therefore, important to also introduce students to the essence of mathematical and computational modeling and its central role.

### 2.1 Course structure and components

#### 2.1.1 On models and modeling

##### 2.1.1.1 Objective and key concepts

The course begins with an introduction of basic, high-level, contrasting key concepts:

1. Complicated vs. complex systems
2. Predictability vs. emergence
3. Engineered vs. biological systems
4. Hypothesis vs. data-driven research
5. Reductionism vs. holistic approaches
6. Theoretical vs. Mathematical vs. computational approaches (see Appendix)

The discussion subsequently introduces the need for mathematical and computational approaches. Since these assume the existence of a model, the discussion starts with the following working definition “a model is a reduced representation of physical reality.” The definition is used to establish that a model depends on.

- Context: Biological, clinical, or mathematical/computational; and
- Scope: Resolution, appropriateness, feasibility, reproducibility, relevance, and translational potential

The discussion emphasizes the role of model systems (i.e., how the physical reality is observationally approximated, reflecting specific aspects of interest which are monitored and recorded) and system models (i.e., the types and kinds of mathematical and computational abstractions that are developed to represent, approximate and simulate the model system).

**FIGURE 1:**
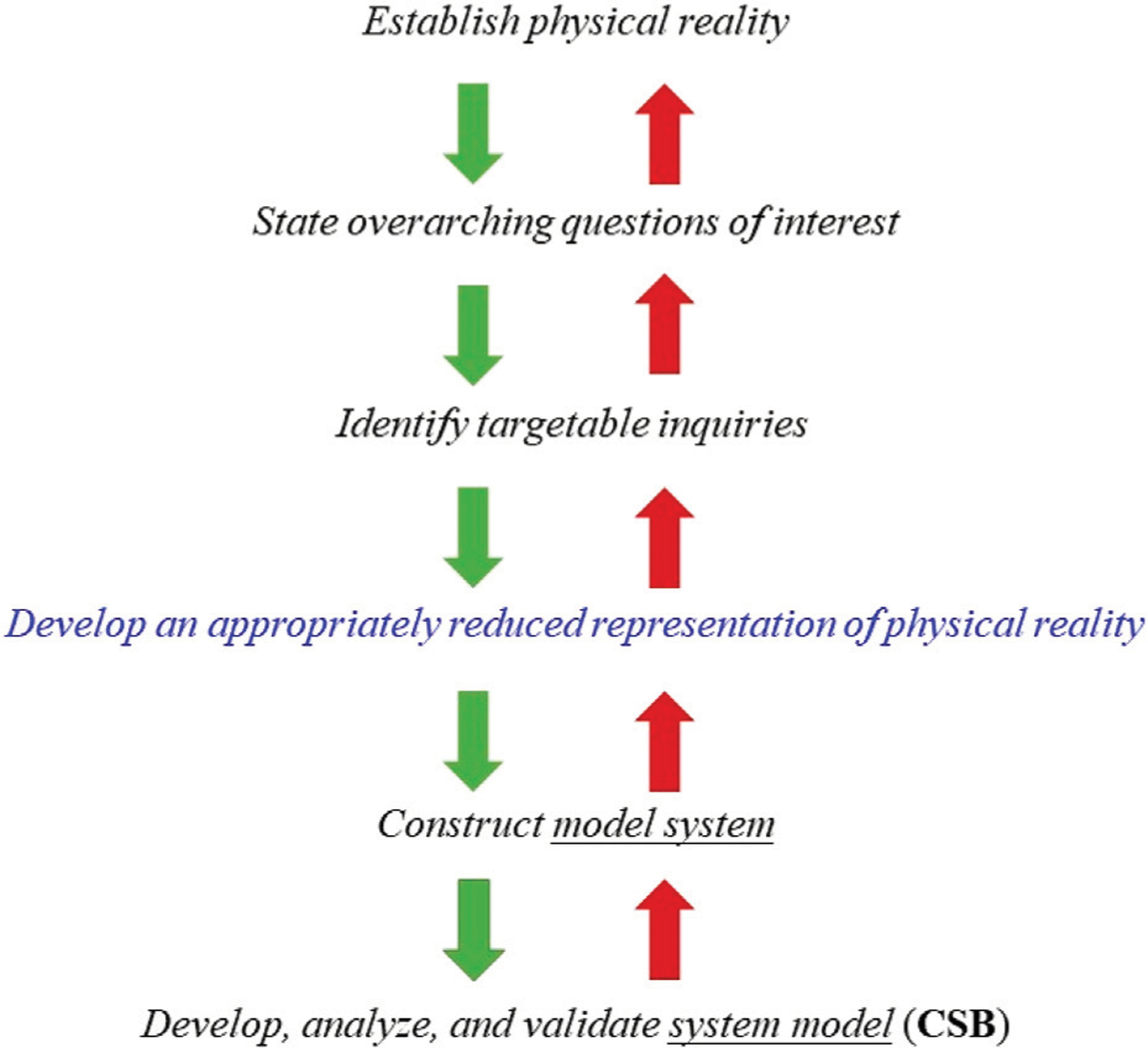
The modeling “continuum” begins with the identification of a physical reality, for example, a disease of interest. Subsequently, relatively smaller but better described sub-tasks are identified which are subsequently decomposed into specific targets for further exploration. Once the latter task has been completed, a reasonable representation of the original system has to be developed and then materialized in the form an *in vitro, ex vivo* or *in vivo* model system which is, at the same time, a reasonable replicate of the physical reality as well as a rendition that can be manipulated, probed, and characterized. So, for instance, the appropriate reduced representation of physical reality can be a particular strain of mouse, whereas the model system can be a collagen-induced arthritis model in mice. The model system and the targeted inquiries are mathematically and computationally described and the resulting the CSB system model is used to explain and/or predict observations obtained with the model system. The validation of the model concerns the comparison of model predictions to available experimental data.

The purpose of the first module is to introduce students to the “modeling continuum”, Figure 1, which sets the process within a context. The continuum encompasses the following steps: 1) Define a physical reality, e.g., the development of human disease; 2) state overarching goals, e.g., reduce tumor growth rate; 3) identify targetable inquiries, e.g., specific molecular pathway, target, or drug molecule; 4) develop an appropriately reduced representation of physical reality, e.g., *in vitro, ex vivo, in vivo* model with specific characteristics; 5) construct a model system, e.g., specific instantiation such as animal model or cell culture with clearly defined properties amenable to measurements as well qualitative and quantitative description; 6) construct the system model, e.g., express important properties of the model system using computational and mathematical approaches in a way that these can reasonably describe the observed response of the model system.

##### 2.1.1.2 Suggested literature

Reading materials include.

1. What is systems biology (Breitling, 2010)
2. The nature of experimental observations (Jose, 2020)
3. Likely pitfalls of systems biology (Joyner and Pedersen, 2011)
4. Mathematical modeling in a clinical setting (Buchman, 2002; Buchman, 2004; Buchman, 2009)
5. The classic work of Claude Bernard (Bernard, 1949) is a reference material of broader interest. A key quote from the book is used as a central motivation, namely, “The application of mathematics to natural phenomena is the aim of all science because phenomenal law should always be mathematically expressed.”

##### 2.1.1.3 Outcome

Enable students to develop a solid understanding the purpose of computational and mathematical modeling, its strengths, and limitations in the context of biological and physiological systems.

#### 2.1.2 Principles of indirect response modeling

##### 2.1.2.1 Objective and key concepts

This module introduces the basic concepts of physicochemical modeling and focuses on the basic principles of indirect response modeling (IRM) as an approach to provide a semi-mechanistic template and formalism for describing the propagation of external signals across complex cascades of signal transduction and transformation. The discussion also involves simple models characteristic of more complex interactions, such as tolerance, rebound, and transit compartment modeling for approximating delayed signal transduction. The IRM concept is introduced alongside a discussion of mass action law to demonstrate how simple rules can enable approximation of, yet to be determined, dynamic interactions. IRM is discussed in the context of modeling the dynamics of biological/biochemical and physiological dynamics, focusing on the ubiquitousness of this modeling approach.

**FIGURE 2:**
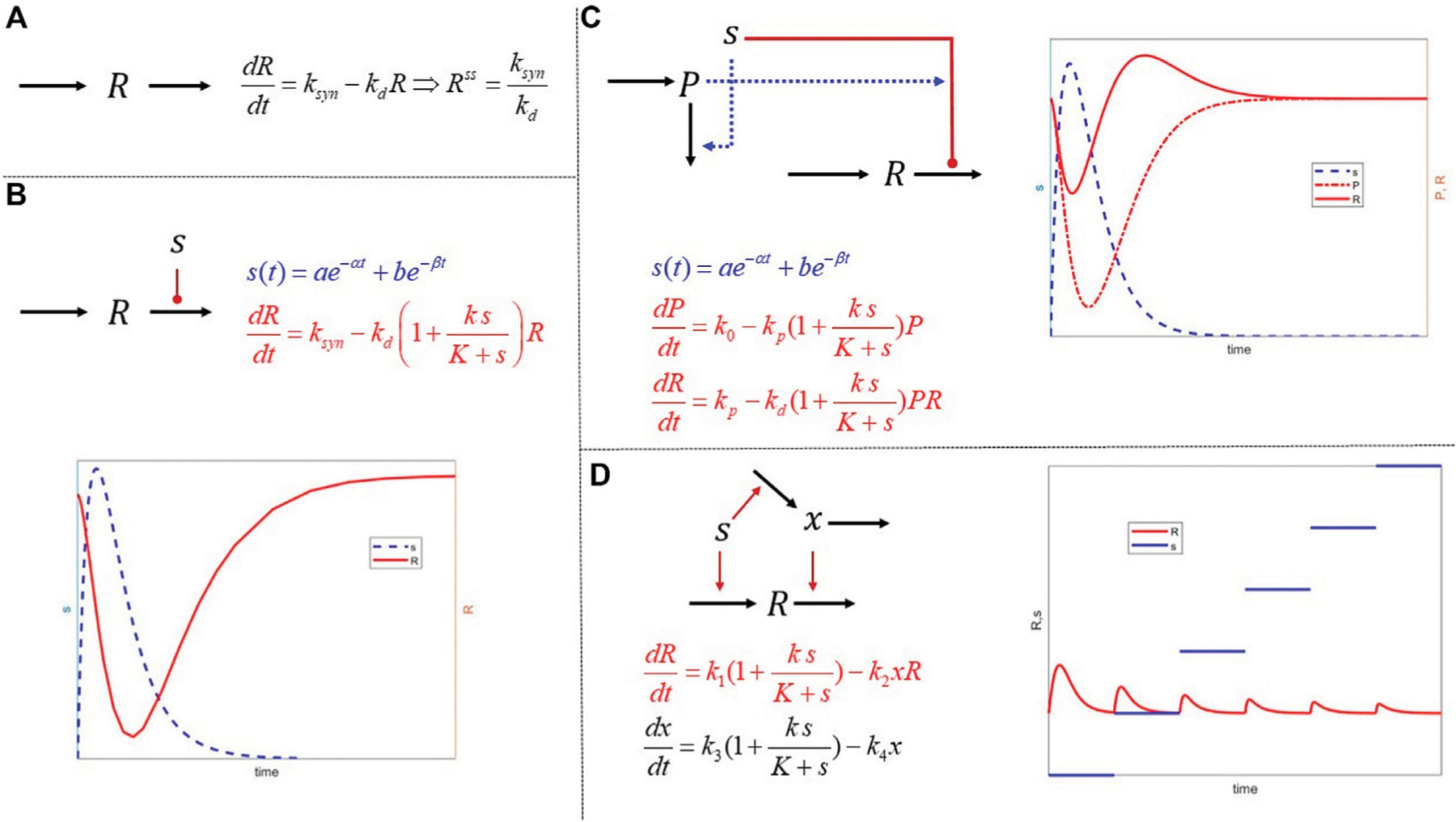
Mathematical structures of increased complexity combing the principles of mass action and IRM. (**A**) The basic dynamics of a physiological or biological response described as the balance of a 0th order synthesis and 1st order degradation. Such models are often used to quantify homeostatic levels of biomarkers; (**B**) external signals act on either the synthesis or the degradation of a response. The hypothesis behind IRM is that although the exact signal transduction may not be known in detail, the effects are approximated using functional forms acting either the synthesis or degradation elements. The function forms themselves can encapsulate more complex phenomena, such as receptor saturation; (**C**) combining simpler structures and establishing interactions among them can induce more complex responses such rebound and memory effects by incorporating second signals regulated by the primary; finally (**D**) tolerance is used as another example of an interesting behavior emerging by combining simple, but cross-regulating, modules. It is important to realize that in all these examples, the mathematical expressions are simple and implement putative hypotheses. As opposed to being mathematical terms of arbitrary complexity leading to complex responses, the dynamics emerge as a result of connectivity of simple elements.

The concept of IRM is introduced as a simple yet robust framework which enables the construction of semi-mechanistic representations in a modular form. These expandable structures enable the formation of a complex network that can exhibit non-intuitive dynamics based on simple components. In Figure 2, we depict a few characteristic structures. The fundamental unit 1) presumes that any response expresses the equilibrium between a 0th-order rate of synthesis and a 1st-order degradation. So, at “steady state,” the response, *R*, system is assumed to exist at a condition such that:

(1) $$\frac{dR}{dt}\;=\;k_{syn}\;-\;k_{d}R\;=\;0\;\to \;R^{ss}\;=\;\frac{k_{syn}}{k_{d}}$$

*R* can describe any physiological response of interest. Any external signal, *s*, acting on the system can impact either the synthesis or degradation processes and does so by inducing or suppressing the synthesis and/or degradation dynamics. The suppression and induction terms can assume functional forms consistent with the hypotheses to be tested. Therefore, the constitutive Eq. 1 takes the form

$$\frac{dR}{dt}\;=\;k_{syn}f\left(s\right)\;-\;k_{d}g\left(s\right)\;R$$

For example, if we assume that the external signal drives some receptor-mediated effect on the system, then

$$f\left(s\right)\;=\;\left(1\;\mp \;\frac{k_{syn.f}s}{K_{syn.f}\;+\;s}\right)$$

$$g\left(s\right)\;=\;\left(1\;\mp \;\frac{k_{deg.g}s}{K_{deg.g}\;+\;s}\right)$$

The signal *s* can be either a constant or a time-dependent entity itself, and, as seen in (b), a transient signal stimulating the degradation of *R*, leads to its reversible suppression.

With these modules as the basis, we can begin to articulate mode complex structures. For example, in 3) we have a typical precursor-mediated response leading to a non-intuitive “rebound” effect in *R*. The network we construct assumes that the signal *s* stimulates the degradation of *R*, but also stimulates the degradation of a precursor, *P*, which also drives the degradation of the response. Even though the constitutive elements are simple, the emergent response is rather interesting as it induces a so-called “rebound” effect with the response *R* being transiently reduced; however, the return the steady state as the external signal clears exhibits a rebound as the precursor gets depleted.

The final example 4) describes a simple tolerance model, whereby the signal *s* induces the synthesis of the response but also drives a secondary signal which induces the degradation of *R*. One can then demonstrate that for this system:

$$\frac{dR}{dt}\;\;=k_{1}\;\left(1+\frac{ks}{K\;+\;s}\right)\;-\;k_{2}xR\;=\;0\;\;\;\;R^{ss\;}=\;\frac{k_{1}k_{4}}{k_{2}k_{3}}\;\;\;\;\;\;\;\;\;\;\;\;\;\;\;\;\;\;\;\;\;\;\;\;\;\;\;\;\;\;\;\;\;\;\;\;\;\;\;\;\;\;\;\;\;\;\;\;\;\;\;\;\;\;\;\;\;\;\;\;\;\;\;\;\;\Rightarrow \;\frac{dx}{dt}\;\;=k_{3}\;\left(1+\frac{ks}{K\;+\;s}\right)\;-\;k_{4}x\;=\;0\;\;\;\;x^{ss\;}=\;\frac{k_{3}}{k_{4}}\left(1\;+\;\frac{ks}{K\;+\;s}\right)$$

Running a simple experiment in which a constant signal *s* is presented to the system over a period of time, and subsequently, it is increased, we notice that the eventual steady state of the system is independent of the magnitude of the signal. This collection of simple modules enables the description of a system that eventually develops tolerance.

This module aims to provide students with basic concepts of kinetics and demonstrate how complex dynamics can emerge when simple(r) elements are appropriately connected. This subject will be revisited in subsequent modules of the course.

##### 2.1.2.2 Suggested literature

Reading materials include.

1. Basic principles of physicochemical modeling (Aldridge et al., 2006; Bae et al., 2019)
2. A basic introduction to capacity-limited systems as a fundamental concept guiding biological transformations at all levels (Jusko, 1989)
3. Fundamentals ideas supporting the theory of IRM (Krzyzanski and Jusko, 1997; Krzyzanski and Jusko, 1998; Sharma and Jusko, 1998; Mager et al., 2003)
4. Modeling of physiologic responses using IRM (Jusko and Ko, 1994)

##### 2.1.2.3 Outcome

Enable students to develop the basic foundation of mathematical expressions based on simple rules (mass action, resource-limited kinetics) to describe a wide range of biological and physiological processes.

#### 2.1.3 Pharmacogenomics

##### 2.1.3.1 Objective and key concepts

Pharmacogenomics, loosely described as the study of how drugs impact transcriptional responses, is introduced as a way to incorporate “drugs” (instead of external signals). This module illustrates how physicochemical modeling and IRM structures can be used to start establishing complex structures describing transcriptional, translational, and post-translation effects. The students are introduced to the cascade of events involving.

1. Signal reception
2. Signal transduction
3. Biological response

**FIGURE 3:**
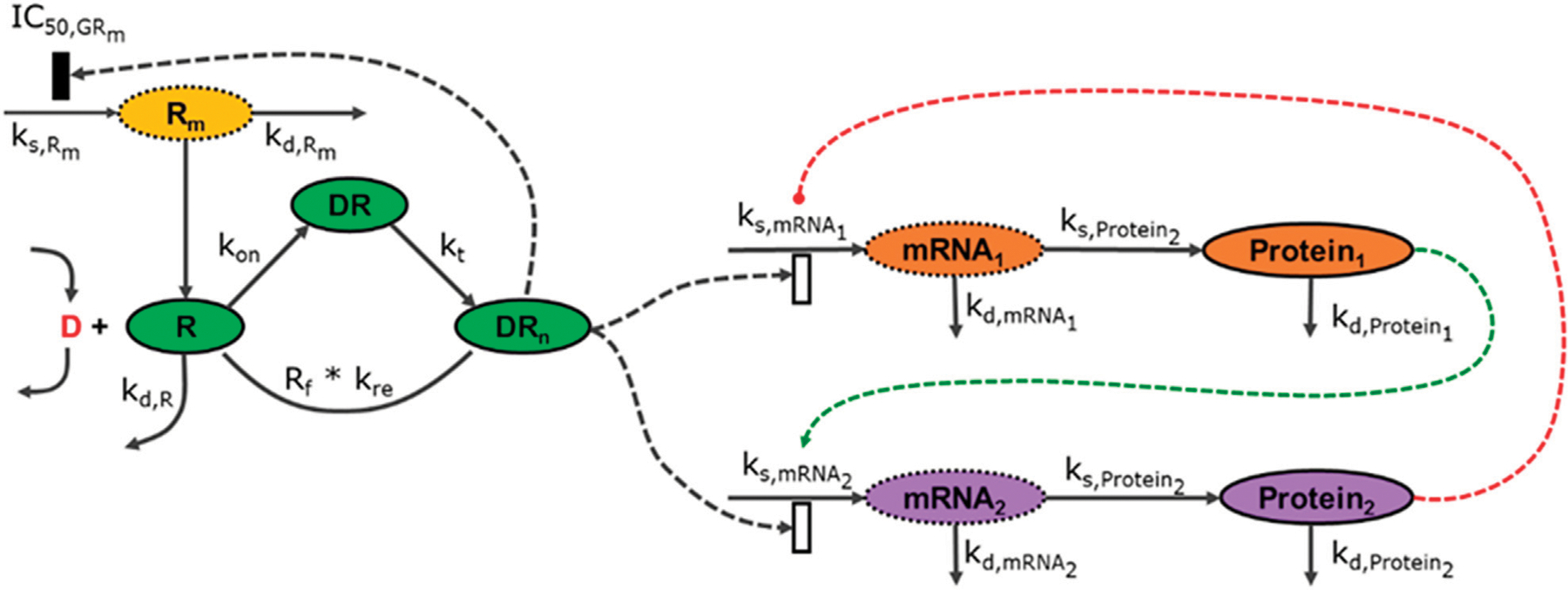
The modalities described in Figure 2 can be connected to an arbitrary degree, linking components across multiple scales. In this illustration, the external signal (***D***) binds to a receptor (***R***) creating a complex (***DR***), which upon translocation to the nucleus gets activated (***DR***_***n***_ ) which subsequnelty regulates the expression of genes (***mRNA***_**1**_, ***mRNA***_**2**_) which upon translation produce proteins (***Protein***_**1**_, ***Protein***_**2**_) which further cross-regulate the system.

Pharmacogenomics introduces high-throughput data (transcriptomic, proteomic and metabolomic data) and demonstrates how multiple data modalities can be connected using mathematical models and structures discussed in the previous (IRM) module, Figure 3.

##### 2.1.3.2 Suggested literature

Reading materials include.

1. Using transcriptomic data to develop pharmacogenomic models (Jin et al., 2003)
2. Development of large models (Ayyar and Jusko, 2020)
3. Model-based integration of -omic data (Kamisoglu et al., 2017; Ayyar et al., 2018)
4. Combining disease models with pharmacologic interactions (Rao et al., 2016b)

By the end of the first three modules, the students would have developed a basic understanding and appreciation for the need for modeling and basic mathematical formalisms, based on ODEs, for expressing the dynamics of biological and physiological transformations.

##### 2.1.3.3 Outcome

Enable students to appreciate the ability of a mathematical or computational model to reflect a quantifiable representation of a biological hypothesis.

#### 2.1.4 The power of feedback and the emergence of ultra-sensitivity, hysteresis, stability, bistability, and periodicity

##### 2.1.4.1 Objective and key concepts

Having established the functional forms combining mass action law and IRM, in this module, we proceed to discuss two critical topics.

**FIGURE 4:**
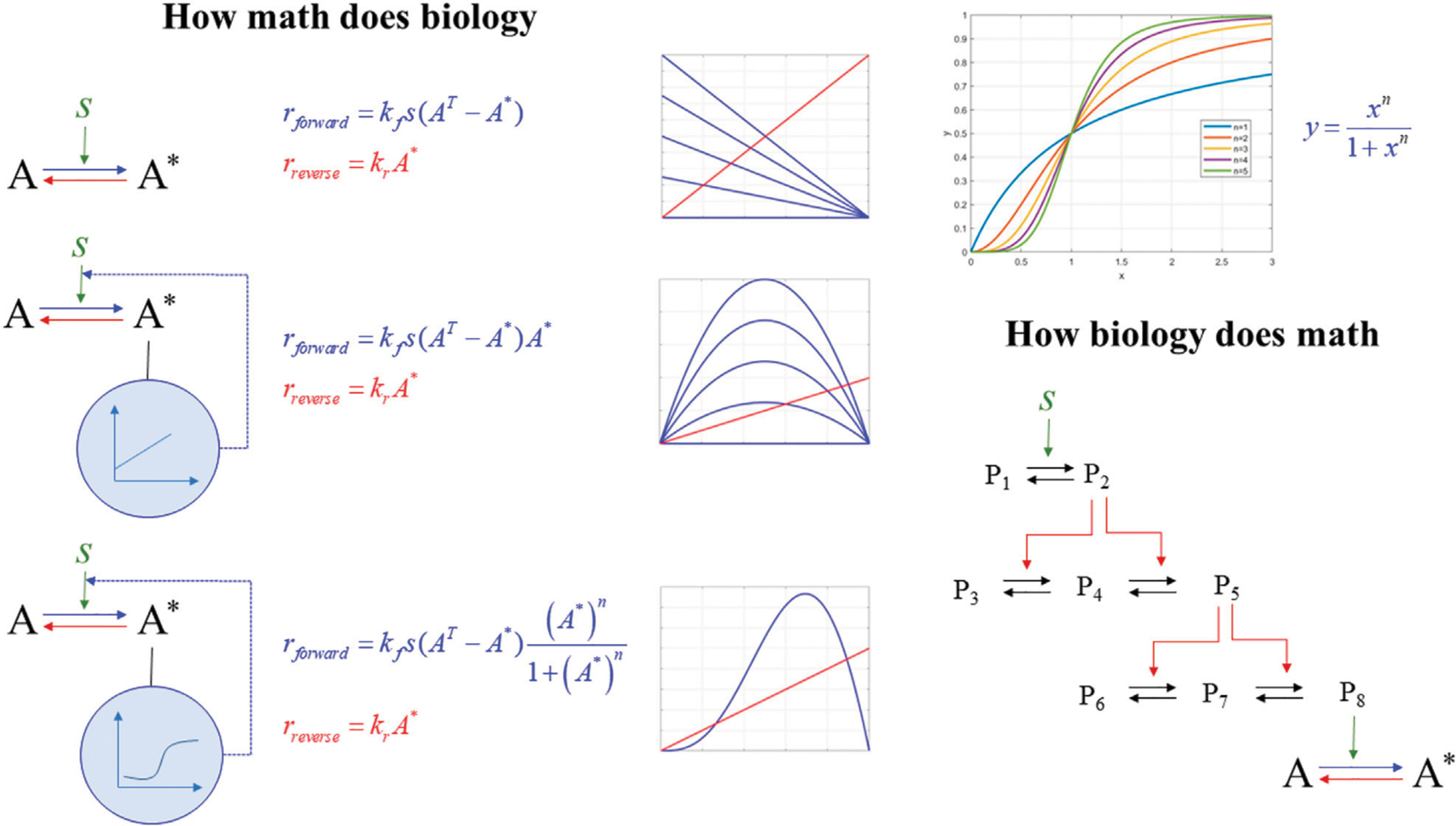
How math does biology vs. how biology does math. (left) the dynamics of an activation (*A* ↔ A*) produces interesting dynamics depending on the existence, and type, of feedback. No feedback (top) produces a system existing in a single steady state depending on the strength of the external signal, *s*; (middle) adding simple linear feedback creates a system with two steady state, one stable and one unstable; whereas (bottom) a Michaelis-Menten (receptor situatable) feedback will produce two stable and one unstable steady state, thus creating the possibility for a bistable, and eventually, oscillating system. Ultra-sensitivity (right top) is mathematically modeled using a Hill-type equation, however, in nature (right bottom) signaling cascades, like the MAPK, exhibiting successive multi-site catalyzed reactions, among others, produce physiological ultra-sensitivity. Simple modeling, using mass action of the multi-site catalyzed systems replicates the dynamics generated by the, non-physical, Hill equation.

- *How math does biology*: We discuss how rewiring seemingly, simple structures and combining basic mathematical modules gives rise to non-obvious dynamics responses. We focus on ultra-sensitivity (how the differential response to external signals can be modulated to become dependent on external signals within narrow ranges), hysteresis (enabling the system to develop memory), and stability and bistability (how a system can exist in several states). This component is essential as it introduces the general student population to novel conceptual and computational concepts (such as sensitivity, hysteresis, and bistability). It discusses how non-intuitive, complex, and emergent properties result from simple, fundamental transformations.
- *How biology does math*: We discuss how nature has evolved signaling structures composed of elementary biological modules described by basic mass action laws. However, the emergent response is highly nonlinear once these modules come together. This discussion is critical to establish the relevance of mathematical formalisms in mirroring biological reality. Signaling cascades lead to hypersensitivity and biostability. The importance of this component is to present students with how nature has evolved mechanisms to support foundational elements, Figure 4.

Particular emphasis is given to feedback and its role in biology and homeostasis (El-Samad, 2021). This module concludes with a discussion of circadian rhythms as a prime example of exciting dynamics (periodicity) emerging through combining the abovementioned elements. This discussion is primarily stimulated through the analysis of the fundamental models of Goodwin and Goldbeter (Goodwin, 1965; Goldbeter, 1995) and expanded to recent models of the hypothalamic-pituitary-adrenal, HPA, axis (Rao and Androulakis, 2019).

##### 2.1.4.2 Suggested literature

Reading materials include.

1. Ultra-sensitivity, bistability, and hysteresis (Ferrell and Ha, 2014a; Ferrell and Ha, 2014b; Ferrell and Ha, 2014c)
2. Signal amplification in molecular networks (Zhang et al., 2013)
3. Design principles of biological control systems (Tyson et al., 2003)
4. Design principles of biochemical oscillators (Novak and Tyson, 2008)
5. Model circadian oscillators (Goodwin, 1965; Goldbeter, 1995)

##### 2.1.4.3 Outcome

Introduce students to mathematical formalisms composed of simple computational elements that describe complex, nonlinear dynamics observed in biological and physiological systems. Demonstrate how the assembly of such units replicates biological reality. Emphasize that emergence is the result of appropriately connected simplicity.

#### 2.1.5 Optimization, parameter estimation, and model assessment

##### 2.1.5.1 Objective and key concepts

Parameter estimation sits at the core of model building in CSB! (Myers et al., 2019). In this module, the students are introduced to basic optimization concepts, such as types of formulations, unconstrained and constrained optimization, optimality criteria, essentials of gradient decent and stochastic optimization, sensitivity analysis, the multiplicity of solutions, global vs. local minima, parameter estimation in dynamic systems, uncertainty, and flexibility analysis. The students are exposed to the core, underlying principles of standard parameter estimation problems whose solution is required for quantifying, i.e., deciphering parameters, in the models described earlier. The discussion emphasizes the difficulties associated with parameter estimation in large CSB models, particularly as it relates to challenges associated with such models, namely, lack of model completeness and lack of reliable data. Finally, the course discusses the important issue of model assessment: deciding on the appropriateness of the model. In addition to introducing basic concepts for model validation, the discussion further emphasizes the broad nature and scope of CSB models and the complexities in introduces related to assessing the appropriateness.

##### 2.1.5.2 Suggested literature

Reading materials include.

1. Parameter estimation in systems biology (Poyton et al., 2006; Gutenkunst et al., 2007; Ashyraliyev et al., 2009; Liepe et al., 2014; Meyer et al., 2014; Degasperi et al., 2017)
2. The intrinsic difficulty of estimating parameters in biology (Gutenkunst et al., 2007)
3. An enormous number of excellent optimization textbooks exist. The classic textbook (Gill et al., 1981) is suggested as a gentle introduction to basic concepts of optimization
4. On model assessment (Androulakis, 2022)

##### 2.1.5.3 Outcome

Enable students to understand the optimization foundation of parameter estimation and develop a basic knowledge of the formulation of a parameter estimation problem, focusing on dynamic systems.

#### 2.1.6 Elements of machine learning

##### 2.1.6.1 Objective and key concepts

The discussion up to this point is based on a fundamental premise: biological hypotheses exist or can be speculated. Using basic principles (mass action law, indirect response), these hypotheses are expressed using mathematical expressions. Subsequently, computational are performed containing the model(s) in the context of (often limited) available data. Machine learning is discussed in the context of assisting model development when hypotheses can be readily formed, but a significant amount of (diverse) data exist. Therefore, closing the gap due to the lack of hypotheses can be accomplished by “intelligent” analyses of the data.

Machine learning is an enormous topic, and this (brief) module aims to make students aware of the possibilities. Topics covered include:

- Supervised (classification) and unsupervised (clustering) methods
- Support vector machines
- Data reduction (PCA, muti-dimensional scaling)
- Feature selection
- Universal function approximations (neural networks)

##### 2.1.6.2 Suggested literature

Reading materials include (the literature is enormous, and it is not the purpose of this module to cover the entire literature)

1. Machine learning fundamentals (Fukunaga, 1990) and recent developments in the context of machine learning (Sing, 2022)
2. The role of machine learning in assisting model building (Benzekry, 2020; Zhang et al., 2022)
3. Machine learning applications in the fields of systems biology and pharmacology (Nguyen et al., 2009; Yang et al., 2018; Gaw et al., 2019; Guthrie et al., 2019; McComb et al., 2021)

##### 2.1.6.3 Outcome

Expose students to the challenges and opportunities associated with assembling and analyzing vast, diverse amounts of data in the absence of underlying principles and discuss how proper and systematic analyses can lead to novel insights and testable hypotheses.

#### 2.1.7 Getting started with quantitative systems pharmacology

##### 2.1.7.1 Objective and key concepts

The last module of the course exposes students to a translational aspect of CSB, which discusses the principles mentioned above in the context of disease and pharmacological treatments. This integrated view defines what is now commonly referred to as Quantitative Systems Pharmacology (QSP). While developing the course, we felt that this conclusion to the course would be critical for Biomedical Engineering students since it is very likely that most of them will eventually be employed by the pharmaceutical industry. Therefore, exposure to the subject and understanding the basic modeling and computational principles would provide a significant competitive advantage. The field of QSP is quite broad (Androulakis, 2015; Androulakis, 2016; Scheff et al., 2016; Hartmanshenn et al., 2018; Putnins and Androulakis, 2019), and impossible to cover the range of applications. Through discussion of case studies (involving invited speakers), the module aims to bring to the forefront the translational opportunities CSB/QSP creates, most notably in generating a virtual cohort of patients/subjects enabling the *in silico* testing of hypotheses in populations. In this module, data-driven (Boolean) and hybrid data/mechanistic (agent-based) models are also introduced.

##### 2.1.7.2 Suggested literature

Reading materials include.

1. QSP methodology toolboxes (Cheng et al., 2017; Ribba et al., 2017; Ermakov et al., 2019; Kirouac et al., 2019; Chae, 2020; Derbalah et al., 2020; Hosseini et al., 2020; Gong et al., 2021)
2. QSP applications (Rieger and Musante, 2016; Stein and Looby, 2018)
3. QSP applications (Bai et al., 2014; Gadkar et al., 2014; Gadkar et al., 2015; Lu et al., 2015; Allen et al., 2016; Rieger and Musante, 2016)
4. Translational aspects of mathematical models (Foteinou et al., 2009; Foteinou et al., 2011)
5. Boolean (Putnins and Androulakis, 2019; Putnins and Androulakis, 2021; Putnins et al., 2022) and agent-based (Dong et al., 2010; Nguyen et al., 2013) modeling.

##### 2.1.7.3 Outcome

The final module of the course aims to discuss how the concepts discussed throughout the course materialize in the context of QSP, which defines a field with definite and clear translational potential.

#### 2.1.8 Capstone project

##### 2.1.8.1 Objective and key concepts

The course concludes with a final project in which the students, working in small groups, are assigned a paper and are requested to:

- Describe the overarching problem discussed in the article, including motivation and a brief literature review
- Describe the basic biological concepts that are modeled, the mathematical model(s) and approaches(s) used, and how these connect to concepts discussed in class
- To the best of their abilities; students should reproduce as many of the results in the paper as possible. If they fail to do so, they need to discuss why
- Prepare a presentation (not to exceed 15 min) describing the above points

The primary purpose of these discussions is for the students to 1) be exposed to a wide variety of problems; 2) see how modeling is used; 3) appreciate that building and running a mathematical model—even if everything is given is not as easy as one may think; and 4) realize the fundamentally interdisciplinary nature of CSB! The project allows the class to initiate a discussion on model reproducibility and the need for establishing repositories (such as GitHub) and methods for assessing published computational models.

##### 2.1.8.2 Sample projects

The topics vary each year and are selected to cover a wide range of issues, serving the additional purpose of exposing students to various problems, questions, and approaches. Projects in years included, among others:

1. Modeling genetic networks with noisy and varied experimental data: the circadian clock in *Arabidopsis thaliana* (Locke et al., 2005)
2. Mathematical modeling of p53 pulses in G2 phase with DBA damage (Zhang et al., 2014)
3. Recurrent initiation: A mechanism for triggering p53 pulses in response to DNA damage (Batchelor et al., 2008)
4. Modeling a simplified regulatory system of blood glucose (Liu and Tang, 2008)
5. Autoinhibition with transcriptional delay: A simple mechanism for the zebrafish somitogenesis oscillator (Lewis, 2003)
6. Mathematical model of NF-κB regulatory module (Lipniacki et al., 2004)
7. Control of neuronal excitability by calcium-binding proteins (Bischop et al., 2012)
8. A skeleton model for the network of cyclin-dependent kinases driving the mammalian cell cycle (Gerard and Goldbeter, 2011)
9. A new model for the HPA axis explains the dysregulation of stress hormones on the timescale of weeks (Karin et al., 2020)
10. Modeling the COVID-19 epidemic and implementation of population-wide interventions in Italy (Giordano et al., 2020)
11. Modeling cortisol dynamics in the neuroendocrine axis distinguishes depression and post-traumatic stress disorder (PTSD) in humans (Sriram et al., 2012)
12. Modeling sex differences in pharmacokinetics, pharmacodynamics, and disease progression effects of naproxen in rats with collagen-induced arthritis (Li et al., 2017)

##### 2.1.8.3 Outcome

The project aims to be a truly integrative experience at many levels: 1) By forming groups assigned randomly, students learn how to coordinate and organize; 2) by assigning topics randomly, students are challenged to move away from their personal comfort areas while delving into new areas; 3) the project requires students to address a range of questions, from discussing the overarching need to understanding the basic biology, to translating physiological reality to mathematical formalisms to eventually using computational methods and numerical analysis to express system dynamics, thus enabling students to appreciate all the cross-disciplinary steps involved in developing and supporting a computational activity; finally 4) students appreciate the importance of supporting a computational model as well as its value-added. Finally, the students receive only high-level guidance from the instructor, as the aim is precisely to have students appreciate the complexities associated with the process of developing a computational analogue. Therefore, while small in scale, the capstone projects aim at exposing students to the entire “supply chain” of developing a CSB model! Students are expected to appreciate the process of developing and using judiciously a computational model, maybe more so that the specific technicalities involved in its development.

### 2.1.9 Computational sources and framework

A CSB course is, by definition, a computational course. The capstone project serves the purpose of enabling students to implement numerical simulations. In this course, students can use an environment that better suits their needs and capabilities. Historically, at our institution, MATLAB is the platform of choice and is the default in Biomedical Engineering. Therefore, students usually opt to develop their codes in that environment. Other options could include *R* or *Python*; however, since it is not the purpose of this course to introduce a new programming platform, we do not impose constraints in that respect. One could envision tailoring the course to *R* or *Python*, but that assumes that either the students are familiar and comfortable with these options or that part of the course is dedicated to developing that skill. We felt that class time was better spent on discussing the modeling component rather than programming languages.

### 2.1.10 Course assessment

Student assessment can be conducted in several ways. In this implementation, a student’s grade depends on a mid-term exam focusing on the material described in Sections 2.1.1–2.1.5 and the capstone project (the Supplementary Materials section provides samples of exam questions). The capstone project is conducted in groups, with each group made up of 3–4 students to achieve a reasonable balance. Students are expected to submit a written report and deliver a 30 min class presentation followed by a Q/A session in which the presenters pose the class questions. The project is expected to include the following components:

- Describe the overarching question, including motivation and appropriate literature review
- Describe the basic biological concepts that needed further assessment and the necessary mathematical formalisms for their representations.
- Reasonably reproduce the computational results presented in the paper. Students are expected to make a serious effort in these directions and should discuss what worked and, more importantly, where they failed to reproduce published results. For the latter, students are expected to describe possible causes, including lack of data, parameter values, or incomplete descriptions.
- Finally, the students are required to critically assess the model’s value and the results and elaborate on possible deficiencies and/or suggested improvements.

## 3 Concluding remarks

In medicine, there is a clear distinction between a disease and a syndrome. The former has a clear and defining cause, distinguishing symptoms, and, usually, a characteristic treatment. A syndrome, on the other hand, is the manifestation of a group of symptoms that may not always have a definite cause. Sepsis, for instance, is a classic example of a syndrome that is described by a set of physiological, biological, and biochemical abnormalities resulting from a dysregulated response to infection. A disease like conjunctivitis (pink eye), on the other, had an unambiguous description (inflammation or infection of the outer membrane of the eyeball and inner eyelid. It has a clear cause (viral or bacterial infection) and specific symptoms (redness, itching, and tearing) and can be treated with the use of antihistamines.

It may not sound very appropriate, but to some extent, discipline-specific courses are more like “diseases,” whereas “integrated or integrative” topics, such as CSB, are more like “syndromes.” When discussing CSB it is hard to define the content uniquely. In our embodiment, for example, we do not discuss the vast area of metabolic engineering or metabolic and flux balance analysis, among others. This is not because they are not relevant or essential but because we described one attempt to approach the question. Much like diseases vs. syndromes, the latter are not amenable to well-defined treatments and exact regiments. Instead, one looks at the history and background of the patient, other biases which may emerge due to circumstances, and the type of expertise available at the time of treatment.

Similarly, when developing a CSB biology curriculum, one needs to consider the “patient,” i.e., the student, and the “health care provider,” i.e., the department. In that respect, we presented a plan developed with primarily biomedical engineers in mind. The development is built on the background, expertise, and expected employment opportunities of a typical biomedical engineer at the undergraduate level. On the other hand, the course’s instructor also brings their own biases, perspectives, and experiences which act, to some extent, as filters prioritizing components eventually forming the broader topic. In that respect, we felt that a CSB with “an eye” on QSP would provide students with defined expertise. Last but not least, CSB courses can play a significant role in increasing bioengineering student awareness on the importance of theory, modeling, and computation in an era when quantitative skills dwindle to dangerous levels (Gatchell and Linsenmeier, 2014).

In conclusion, we aimed to present one approach to CSB while emphasizing that the beauty of the field is precisely its richness. CSB cannot, and should not, be uniquely defined. Instead, it establishes a continuum of approaches, a syndrome, and the more avenues we create, the more significant its impact.

## Supplementary Material

2

## Data Availability

The original contributions presented in the study are included in the article/Supplementary Material, further inquiries can be directed to the corresponding author.

## Footnotes

1 Taken from: https://www.bme.ufl.edu/course/biomedical-transport-phenomena-bme-4632/

2 Taken from: https://cbe.engineering.uiowa.edu/file/cbe5115-transport-phenomena-syllabus

3 We draw a distinction between systems biology and computational (or quantitative) systems biology, but we will talk more about that later.

4
https://irp.nih.gov/catalyst/v19i6/systems-biology-as-defined-by-nih

5
https://isbscience.org/about/what-is-systems-biology/

6 We do not draw a distinction between computational systems biology and quantitative systems biology. We assume that the two (computational and quantitative) can be used interchangeably in this context and that both imply the development of mathematical or computational models for analyzing and interpreting systems biology data. For consistency we are using the term computational systems biology (CSB).

7 Please, see appendix

8
https://www.fda.gov/drugs/development-resources/model-informed-drug-development-pilot-program

9 We sincerely apologize that we cannot provide a comprehensive list of systems biology textbooks. This was only a practical limitation, and the selection does not reflect bias of any kind.

10
https://en.wikipedia.org/wiki/Blind_men_and_an_elephant

## Appendix A:

In the manuscript and the course, we attempt to distinguish between three types of modeling: theoretical, mathematical, and computational. The distinction is methodologically important:

A theoretical model is “conceptual” and defines expected boundaries. A very nice illustration of this type of approach is presented in (Tyson et al., 2003) (Figures 1, 2 therein) or the various models described in (Ferrell and Ha, 2014c) (some depicted in Figure 4 of this manuscript) discussing the minimal structural characteristics required so that a model can exhibit a stable steady state, or saturation, or adaptation, or multiplicity of steady states, or periodic dynamics etc. So, a theoretical model establishes boundaries. Theoretical models tend to be minimalistic.

A mathematical model builds on the theoretical model by expanding critical components of a minimalistic theoretical representation. An excellent illustration is how the basic Goodwin oscillator (a prototypical theoretical model for generating sustained oscillation) is used as the foundation for building more complex models describing biological circadian oscillators (Goldbeter, 1995).

A computational model focused on extensive data analysis using (mostly) black box approaches to replicate the observed dynamics. Indeed, they can be based on theoretical or mathematical foundations, but the emphasis is on computational methodologies (Putnins and Androulakis, 2019; Putnins et al., 2022). A neural network is a typical example of a computational model (Yazdani et al., 2020; Przedborski et al., 2021).
