# Supplementary material for: Teaching computational systems biology with an eye on quantitative systems pharmacology at the undergraduate level: Why do it, who would take it, and what should we teach?
Source: Front Syst Biol. Author manuscript; Available in PMC 2023 Mar 1. (PMC9977321; doi:10.3389/fsysb.2022.1044281)

Supplementary Material

# Sample exam problems

**Problem 1:** You conduct an animal experiment which involves the continuous infusion of a drug such that its concentration remains constant leading to an increase of the animal’s heart rate (HR). You record the data shown in the figure bellow

If you assume a simple response model for heart rate and that the heart rate is indirectly affected by the drug, does the drug act by:

(a)... indirectly inhibiting the rate of HR production

(b)… indirectly stimulating the rate of HR degradation

(c)… both

(d)… neither

Given your answer in the previous question explain what you identified as the most likely mechanism of the (indirect) effects of the drug on HR

**Problem 2:** You are given the network shown below.

1. Assume the SIMPLEST possible dynamic model describing the liner induction of the rate of synthesis of R and X by the signal S and the simultaneous induction of the rate of degradation of R by X. S is assumed to be continuously present in the system at a constant level. Derive the steady state values of X and R as a function of S.
2. Assume that the activation of the synthesis of X by S is described by the simple M-M type of expression $\frac{S}{S+1}$ while everything else remains the in (2a). Derive the steady state expressions for R and X as a function of S in this case
3. Compare the results of (a) and (b) and explain the differences, if any


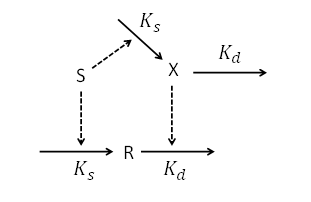


**Problem 3:** The simple network shown below depicts the phosphorylation/de-phopshorylation of P via the assistance of a kinase, K. You can assume that the total amount of the protein is constant ($P^{tot})$:

1. Derive the forward (phosphorylation) and reverse (de-phosphorylation) rate equations assuming simple mass action kinetics, linear kinase effects and linear feedback dynamics
2. Depict graphically the two rates as a function of the amount of phosphorylated protein; identify and depict the possible steady states and discuss their stability


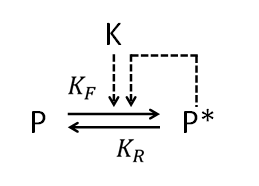


**Problem 4:** The network at the top represents a basic *antagonist-dependent* tolerance model, whereas the network at the bottom represents a typical *precursor-dependent, rebound and tolerance* model.


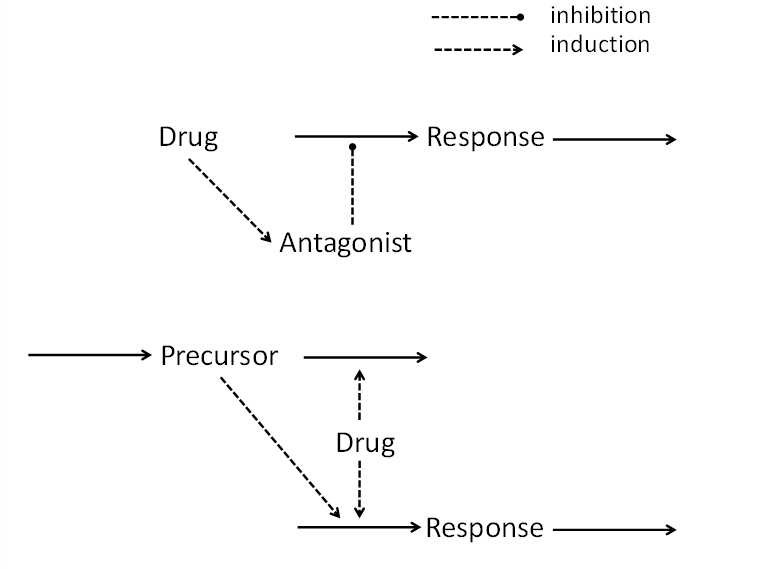


1. Write the basic equations describing the dynamics of the antagonist model and explain how it manifests tolerance using the example of two successive acute stimulations
2. Write the basic equations describing the dynamics of the precursor-dependent model and explain how it manifests its rebound and tolerance effects using the example of two successive acute stimulations

**Problem 5:** The network bellow depicts the transcription of gene *m*, its translation to protein P and its subsequent phosphorylation to P*. The phosphorylated form of the protein translocates to the nucleus where it inhibits the rate of transcription of *m* in Hill-type way.

1. Derive the dynamic model describing the kinetics of the system – clearly identify the parameters used in your model equations
2. Explain the origins of the oscillations this system can potentially exhibit
3. Explain how increasing the rate of “*transfer”* will impact the period of the oscillations


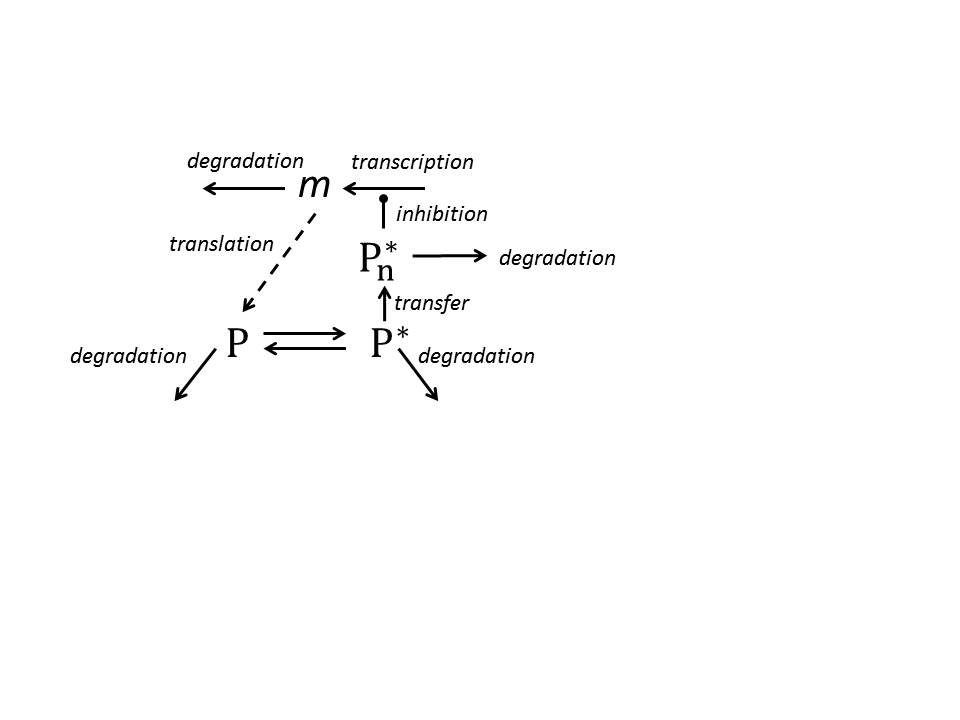


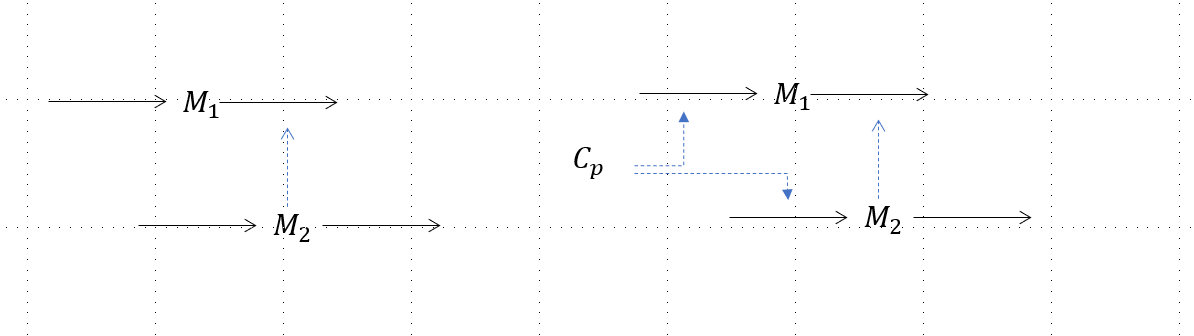
**Problem 6:** You are given the network shown in the figure (LEFT side). For each of the components, you can assume the “standard” 0^th^ order synthesis, and 1^st^ order degradation” kinetics. The “dashed” lines imply an indirect stimulation of the degradation of $M_{1}$ by $M_{2}$. Please note that this stimulation a) is linear; and b) if $M_{2}=0$ the degradation of $M_{1}$ is not 0. This small network (LEFT side) exists in a steady state. The introduction of a drug drives the synthesis of both $M_{1},M_{2}$ in an indirect, linear way (RIGHT side). You are given that the rate constant of synthesis for both $M_{1}, M_{2}$ is 0.3 and the rate constant of degradation for both $M_{1}, M_{2}$ is 0.2. You can assume that any constants associated with indirect response terms are 1.

A drug is administered in two different ways: 1) as a bolus injection in which case its concentration as a function of time is given by $C_{p}\left( t \right)=C_{p0}*\left( e^{-k_{1}t}-e^{-k_{2}t} \right), k_{1}=0.5, k_{2}1.0, C_{p0}=1$; and 2) as a continuous infusion in which case its concentration as a function of time is given by $C_{p}\left( t \right)=C_{p0}$

1. Write the differential equations for $\frac{dM_{1}}{dt},\frac{dM_{2}}{dt}$ in the presence of the drug and determine the proper initial conditions for $M_{1},M_{2}$
2. Develop a MATLAB code (or two …) to generate and plot the profile of $M_{1}(t)$ for a) the bolus injection and b) the constant infusion of the drug. Consider a time horizon of 100 time units which should be enough for the system to reach steady state in either case
3. Explain the differences you observe in the two steady states

**Problem 7:** Methylprednisolone (D) acts by binding its receptor (R) creating a complex (DR) which then translocates into the nucleus and is activated (DRN). The activated complex stimulates the expression a target enzyme (TAT) in a linear manner:


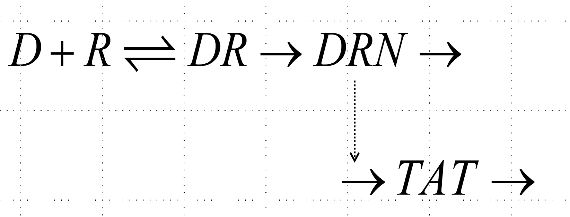


The drug is administered as bolus injection in which case its concentration as a function of time is given by $D=C_{1}e^{-\lambda_{1}t}+ C_{2}e^{-\lambda_{2}t}$. The amount of DR is given by $DR=\frac{B_{max} D_{C}}{K_{D}+D_{C}}$ whereas the kinetics of DRN is given by $\frac{dDRN}{dt}=\frac{DR}{\tau_{1}}-\frac{DRN}{\tau_{2}}$. You are given the following parameter values:

C1 = 39.1

C2 = 12.7

$\lambda_{1}$ = 7.54

$\lambda_{2}$= 1.20

Bmax = 540.7

KD = 2.24

$\tau_{1}$ = 1.53

$\tau_{2}$ = 0.558

The dynamics of TAT, in the absence of drug, follows a basic response expression with

$k_{deg}$ = 0.383;

$TAT_{0}$ = 0.09;

The stimulatory constant for TAT is S = 0.0287.

1. What is the $k_{synthesis}$ of TAT
2. Write a MATLAB code to generate the plot TAT versus time over a period of 60 time units following the injection of methylprednisolone
3. Repeat the calculations assuming a constant infusion of methylprednisolone such that D = 40 units and explain the differences between the two cases

**Problem 8:** Diindolylmethane (DIM) is a natural product abundant in vegetables such as broccoli, cabbage and cauliflower. Animal studies have shown that DIM can reduce tumor growth. To study its effect, male rats were intravenously administered DIM in the form of a single bolus injection. Blood samples were collected over time and the relative expression of various genes, including *nqo1*, was recorded as shown on the figure on the right. Using an indirect response model, suggest and justify a likely mode of action. Derive a differential equation likely describing the dynamics of the mRNA of *nqo1* assuming the indirect effect of DIM on the transcription of *nqo1* is mediated via a saturable receptor.


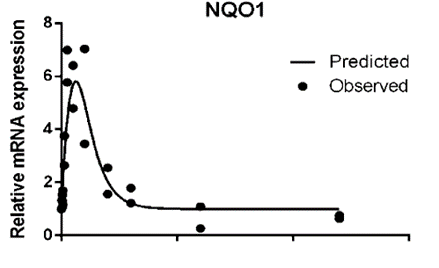

Supplement: 2 [file NIHMS1866247-supplement-2.docx]
